# Genome instability and loss of protein homeostasis: converging paths to neurodegeneration?

**DOI:** 10.1098/rsob.200296

**Published:** 2021-04-21

**Authors:** Anna Ainslie, Wouter Huiting, Lara Barazzuol, Steven Bergink

**Affiliations:** ^1^ Department of Biomedical Sciences of Cells and Systems, University Medical Center Groningen, University of Groningen, Antonius Deusinglaan 1, 9713 AV, Groningen, The Netherlands; ^2^ Department of Radiation Oncology, University Medical Center Groningen, University of Groningen, Antonius Deusinglaan 1, 9713 AV, Groningen, The Netherlands

**Keywords:** neurodegeneration, DNA damage, genome stability, protein homeostasis, protein aggregation

## Abstract

Genome instability and loss of protein homeostasis are hallmark events of age-related diseases that include neurodegeneration. Several neurodegenerative diseases, such as Alzheimer's disease, Parkinson's disease, Huntington's disease and amyotrophic lateral sclerosis are characterized by protein aggregation, while an impaired DNA damage response (DDR) as in many genetic DNA repair disorders leads to pronounced neuropathological features. It remains unclear to what degree these cellular events interconnect with each other in the development of neurological diseases. This review highlights how the loss of protein homeostasis and genome instability influence one other. We will discuss studies that illustrate this connection. DNA damage contributes to many neurodegenerative diseases, as shown by an increased level of DNA damage in patients, possibly due to the effects of protein aggregates on chromatin, the sequestration of DNA repair proteins and novel putative DNA repair functions. Conversely, genome stability is also important for protein homeostasis. For example, gene copy number variations and the loss of key DDR components can lead to marked proteotoxic stress. An improved understanding of how protein homeostasis and genome stability are mechanistically connected is needed and promises to lead to the development of novel therapeutic interventions.

## Graphical abstract

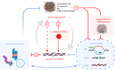

## 1. Introduction

Age-related diseases, such as neurodegeneration and cancer, are on the rise due to an increasingly ageing population. It is estimated that the proportion of people over 60 years of age will double by 2050 [1]. Despite both being associated with age, the underlying mechanisms driving pathogenesis in neurodegenerative diseases and cancer are thought to differ greatly.

For instance, the loss of protein homeostasis is key to the pathogenesis of many neurodegenerative diseases [2,3], illustrated by the presence of protein aggregates in the affected brain areas of patients [4]. Protein homeostasis is maintained via protein quality control (PQC), which coordinates three major facets of protein fate: synthesis, folding and conformational maintenance, and degradation, as described in box 1 and reviewed elsewhere [5–7]. These phases are often overlapping; for example, folding or degradation may happen co-translationally [8].

Box 1. **Protein quality control in human cells.**

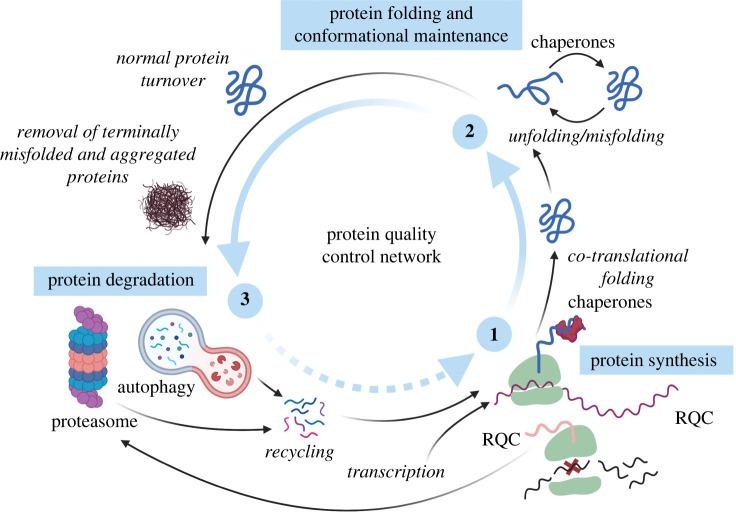

To maintain protein homeostasis in the complex and extremely crowded intracellular milieu, cells rely on the concerted action of molecular chaperones, synthesis regulators and protein degradation pathways. This system, commonly referred to as the protein quality control (PQC) network, guards proteome balance by surveying and controlling three major facets of protein fate.

**Protein synthesis**

Protein quality control starts at the ribosome, where the faithful translation of genetic information is maintained by ribosome-associated protein quality control (RQC). When a translating ribosome stalls on a faulty messenger RNA, this triggers the dissociation of ribosomal subunits, allowing the removal and subsequent degradation of both the mRNA molecule and the nascent polypeptide chain. Globally, translation is regulated by various signalling pathways. Under cellular stress conditions, these repress global protein synthesis, thus lowering the total protein folding burden. In parallel, they induce the selective translational of stress-responsive proteins required for cell survival.

**Protein folding and conformational maintenance**

Most polypeptides exiting the ribosome tunnel must fold into distinct, three-dimensional conformations to become functionally active proteins. To ensure that the final native state is reached, polypeptide folding generally occurs co-translationally, closely monitored through the concerted action of chain-modifying enzymes, translocation factors, molecular chaperones and other quality control components. This enables the emerging polypeptide to navigate a landscape of metastable folding intermediates, while being shielded from non-productive interactions. The majority of folded proteins are thermodynamically only marginally stable, and thus at a constant risk of becoming destabilized and misfolded, both through internal (mutations, stoichiometric imbalances) and external factors (elevated temperature, oxidative stress, heavy metals). Some proteins even require constant chaperone-surveillance to maintain functionality. When the folding capacity of the PQC network is overwhelmed, misfolded proteins can accumulate and partition into insoluble protein aggregates. However, many destabilized proteins can be refolded, or even disaggregated through chaperone intervention. In the presence of excess misfolded proteins, the transcription of additional PQC network factors is induced to restore protein homeostasis.

**Protein degradation**

When proteins are in excess, no longer needed, or when they misfold but cannot be refolded, they are removed from the proteome and recycled. Most protein degradation takes place via two intracellular proteolytic systems: the ubiquitin-proteasome system (UPS) and the autophagy-lysosomal system. The bulk of protein degradation is executed by the UPS. This is a highly specific pathway that requires target proteins to be tagged with ubiquitin (ubiquitylation) and directing them to the proteasome, where they are unfolded and subsequently degraded. Protein molecules that cannot be turned over by the proteasome, like large protein aggregates, or even entire organelles, are degraded via the autophagy-lysosomal pathway. In this pathway, a double-membrane vesicle engulfs sequestered cargo proteins and then fuses with a lysosome, wherein the inner membrane and cargo are digested. Whereas this system generally acts to degrade bulk cargo, (chaperone-mediated) selective sub-pathways that directly target substrates to lysosome exist as well. Although the UPS and the autophagy-lysosomal system are two distinct pathways, extensive crosstalk allows them to compensate for each other when needed.

Of the multiple hallmarks of cancer, the loss of genome stability—defined as an increase in DNA damage and mutations, as well as structural aberrations—is widely regarded as the most common underlying factor in cancer development [9–11]. Under physiological conditions, genome stability is ensured by the DNA damage response (DDR), which encompasses various DNA repair pathways, cell cycle checkpoint activation and cell death [12–14]. Endogenous and exogenous sources cause different types of DNA damage that are repaired by specific DNA repair pathways, as described in box 2 [13,15,16]. The importance of the DDR is illustrated by the more than 50 human disorders caused by malfunctioning DDR processes [17]. Patients suffering from these rare genetic diseases often have a high predisposition to cancer, as is the case for xeroderma pigmentosum (XP) and Fanconi anaemia (FA). Several DNA repair disorders also lead to neuropathological issues including neurodegeneration, as in the case of ataxia-telangiectasia (AT), ataxia-oculomotor apraxia type 1 (AOA1) and Cockayne syndrome (CS) [18–21]. This suggests that, next to a loss in protein homeostasis, genome instability can also lead to neurodegeneration.

Box 2. **DNA repair pathways of relevance for the brain.**

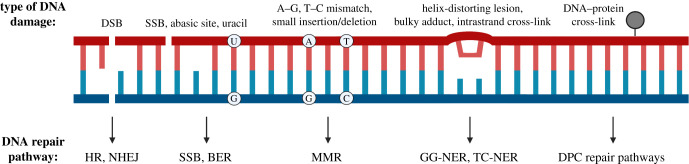

The developing and mature brain is susceptible to various forms of endogenous and exogenous DNA damage, which are repaired by distinct pathways.

**Non-homologous end joining and homologous recombination**

DNA double-strand breaks (DSBs) occur as a result of the breakage of both strands of the DNA. DSBs are repaired by two main pathways, non-homologous end joining (NHEJ) and homologous recombination (HR), whose engagement depends on cell cycle phases and chromatin accessibility. While HR and NHEJ are both available during brain development, in the largely post-mitotic cells of the mature brain DSB repair is largely restricted to NHEJ.

**Base excision repair and single-strand break repair**

Base modifications and single-strand breaks (SSBs), which can occur by direct damage of one DNA strand or as a result of the removal of a damaged base by DNA glycosylases, are common in the mature brain due to oxidative stress. Base modifications and SSBs are repaired by two partially overlapping pathways, base excision repair (BER) and SSB repair pathways, respectively.

**Mismatch repair**

Base mismatches, small insertions and deletions, arising during DNA replication, are repaired by the mismatch repair (MMR) pathway. This type of damage is less common in the largely post-mitotic mature brain.

**Nucleotide excision repair and transcription-coupled nucleotide excision repair**

Structurally unrelated DNA lesions in non-transcribed DNA, such as helix-distorting lesions, bulky chemical adducts and intrastrand cross-links, are repaired by the nucleotide excision repair (NER) pathway. Along with this NER pathway (also called global genome NER pathway (GG-NER)), transcription-coupled NER (TC-NER) can occur in response to long-term stalling of replication. These two pathways are relatively similar and only differ in the way the lesion is detected. In the brain, these lesions are often a consequence of oxidative stress and transcriptional interference.

**DNA–protein cross-link repair pathways**

DNA–protein cross-links (DPCs) constitute a separate class of DNA lesions characterized by protein adducts to DNA caused by endogenous metabolites, other DNA repair intermediates and many chemotherapeutic agents. Repair of these lesions involves three main components based on: the DNA targeted by nucleases; the protein adduct degraded by DNA-dependent proteases and the covalent cross-link between the DNA and the protein adduct that can be hydrolysed by tyrosyl-DNA phosphodiesterases (TDPs). Defects in DPC repair have a detrimental impact on the brain.

Interestingly, increased levels of DNA damage have been observed in many neurodegenerative diseases such as Alzheimer's disease (AD), Huntington's disease (HD), Parkinson's disease (PD) and amyotrophic lateral sclerosis (ALS) [22–30] (table 1). Conversely, PQC components are often upregulated in cancer cells, likely reflecting an adaptive response to a disturbed protein homeostasis [52,53]. This upregulation of PQC capacity mediates cancer cell survival and increased proliferation [54,55]. Thus, although diseases hallmarked by protein aggregation and genome instability are regarded as fundamentally different, they appear to share common underlying mechanisms. Currently, it is unclear how genome instability and protein homeostasis relate, and whether or how this relationship contributes to neurodegeneration.

**Table 1.** Neurodegenerative disease models and DNA damage.

		evidence of increased DNA damage					evidence of increased DDR		evidence of DNA binding
		neutral comet assay	alkaline comet assay	TUNEL assay	DNA electrophoresis	oxidative DNA damage	γ-H2AX	53BP1 and ATM	
AD	amyloid-β OE	mouse dentate gyrus [31]		rat adrenal medulla cells [32]	rat adrenal medulla cells [32]		mouse cortex, hippocampus [31] mouse primary neuronal cells [31]	mouse primary neuronal cells [31]	
	amyloid-β fibrils				fibrillar amyloid-β treatment of naked scDNA [33]				protein–DNA interaction inhibitor ATA prevents DNA strand breakage [33]
	TauKO		mouse cortex [34]	mouse hippocampus [34]			mouse hippocampus [34] mouse cortex and hippocampus, TauKO rescues amyloid-β-induced DNA damage [31]		
	Tau aggregation								aggregated Tau loses its ability to bind to DNA (EMSA and agarose gel retardation assay) [35,36]
	AD patients						AD patient cortex [37,38]	AD patient cortex [37]	
PD	α-synuclein OE		SH-SY5Y cells [39]	mtDNA in mouse brainstem, neocortex, motor neurons [30]	SH-SY5Y cells [39] PD patient derived NPCs [39] α-synuclein treatment of naked scDNA [39]	DNA damage prevented by antioxidant [40]	mouse nigral dopaminergic neurons [40]	mouse nigral dopaminergic neurons [40]	SH-SY5Y cells, α-synuclein ChIP assay [39] α-synuclein and dsDNA, EMSA [41]
	α-synuclein fibrils						mouse nigral dopaminergic neurons after α-synuclein fibril injection [40]	mouse nigral dopaminergic neurons after α-synuclein fibril injection [40]	
	α-synuclein KO	human HAP1 cells [41] mouse cortical neurons [41]					HAP1 cells after bleomycin treatment [41] mouse cortical neurons [41]		
	PD patients					PD patients, elevated levels of OGG1 [42–44]			
ALS	TDP-43 KO	SH-SY5Y cells [45]	SH-SY5Y cells [45]		SH-SY5Y cells [45] *Caenorhabditis elegans* [45]			SH-SY5Y cells [45]	
	SOD1 NLS or mutant		SH-SY5Y cells, NLS-SOD1 rescues DNA damage [46]				SOD1G93A inhibits translocation of HDAC1 to the nucleus [47]		
	FUS KD		SH-SY5Y cells [48] mouse primary cortical neurons [49]						
	mutant FUS		iPSC-derived motor neurons [48]	iPSC-derived motor neurons [48]			mouse cortex and spinal cord [50]		
	ALS patients		ALS patient PBMCs with aggregated SOD1 [46]	ALS patient spinal cord tissues with aggregated TDP-43 [45]	ALS patient spinal cord tissues with aggregated TDP-43 [45]		ALS patient spinal cord tissues with aggregated TDP-43 [45] ALS patients with FUS mutations, motor cortex [49]	ALS patient spinal cord tissues with aggregated TDP-43 [45]	
HD	mutant Htt	rat primary cortical neurons [51]					rat primary cortical neurons [51] mouse striatum [51]		
	HD patients						HD patient neurons [51]		

**Figure 1.**
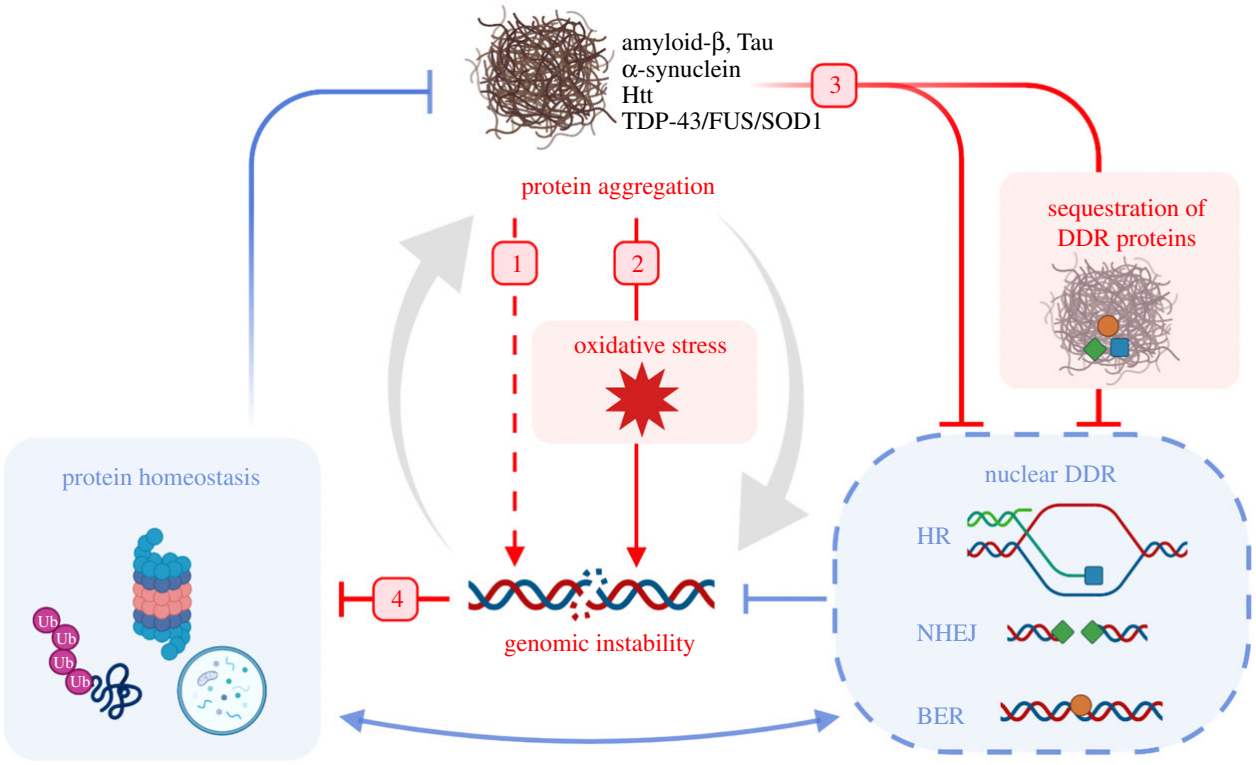
Overview of mechanisms linking the DDR and protein homeostasis that are described in this review. Blue arrows indicate physiological feedback between protein homeostasis, which protects cells from the formation of protein aggregates, and the DDR, which protects cells from genomic instability. Red arrows indicate neurodegenerative disease pathogenesis, as outlined in this review: 1. aggregates can directly cause DSBs; 2. aggregates cause oxidative stress, which induces oxidative DNA damage; 3. aggregates can impair DNA repair; and 4. DNA damage can impair protein homeostasis. The pathological network creates a positive feedback resulting in the accumulation of protein aggregates and genomic instability (large grey arrows). DDR, DNA damage response; DSB, double-strand break; NHEJ, non-homologous end joining; HR, homologous recombination; BER, base excision repair.

Many of the earlier studies linking neurodegeneration and DNA damage were correlative in nature; therefore, the cause-and-effect relationship between DNA damage and protein toxicity has been challenging to elucidate. Recent studies involving animal models of neurodegenerative diseases have begun to shed light on potential mechanisms linking DNA damage and neurodegeneration [56]. This review presents recent evidence of protein aggregates directly causing DNA damage: either double-strand breaks (DSBs) or oxidative DNA damage. Additionally, typical examples that illustrate the mechanisms by which aggregate-forming proteins interfere with DNA repair pathways in different models of neurodegenerative diseases are discussed (figure 1). Lastly, an overview is given of how DNA damage itself, by leading to mistranslation and misfolding of proteins, can perturb protein homeostasis and lead to neurodegeneration.

## 2. DNA damage due to protein aggregation in neurodegenerative diseases

The accumulation of DNA damage has been reported in several neurodegenerative diseases including AD, PD, HD and ALS. Different types of DNA damage have been identified in models of neurodegenerative diseases and post-mortem human brain tissues, mostly DNA DSBs and oxidative DNA damage (figure 1 and table 1). Here, we summarize the evidence that protein aggregates can cause DNA damage either directly or via oxidative stress.

DNA damage can be detected in several different ways, using physical methods that reveal the DNA damage directly such as electrophoresis assays (e.g. pulsed-field gel electrophoresis and neutral comet assays), which are used to detect DSBs, and alkaline comet assays, which can detect both single-strand breaks (SSBs) and DSBs [57]. DNA damage can also be inferred from the detection of activated DDR components, such as the phosphorylation of the histone 2AX (γ-H2AX), which occurs early at sites of DNA damage [58]. The recruitment of other DDR components, such as 53BP1 and ATM, can also be used to indirectly detect DNA damage [59–61]. These last methods do not detect the damage directly, but rather measure a response to DNA damage and can be used as an estimate of the actual amount of DNA damage. Moreover, they do not always accurately report on the type of DNA damage that is present. For example, the phosphorylation of H2AX is well suited as a general marker of genotoxicity but it marks various types of lesions and not only DSBs [62].

### 2.1. Protein aggregates can directly cause DNA double-strand breaks

Several studies have linked AD with a higher frequency of DNA damage markers such as γ-H2AX in affected brain areas [37,38]. Moreover, neural activity-induced DNA damage was exacerbated in an AD mouse model with elevated amyloid-β levels [31]. This finding was supported by experiments using mouse primary neuronal cells, suggesting that amyloid-β oligomers are sufficient to increase the level of DSBs, detected using a neutral comet assay and by staining for DDR components *γ*-H2AX and 53BP1 [31].

It has been proposed that protein aggregates can directly cause DNA breaks. In an early *in vitro* study, incubating naked supercoiled DNA with AD-associated fibrillar amyloid-β protein alone resulted in the formation of circular and linear forms of DNA, suggesting that amyloid-β fibrils are sufficient to induce DNA breaks [33]. Furthermore, treatment with a protein–DNA interaction inhibitor prevented DNA fragmentation, suggesting that DNA break formation is dependent on amyloid-β binding to DNA [33]. However, it is unclear whether amyloid-β has nuclease activity, or whether incubation of naked DNA with any aggregated protein would lead to DNA breaks. High levels of aggregation-prone luciferase also impair the DDR [63], implying that the effects of aggregated amyloid-β are indeed non-specific. Of note, amyloid-β plaques are usually extracellular [64] and therefore not in close proximity to nuclear DNA, making it less likely that this contributes to the clinical manifestations of AD.

A similar direct effect on the integrity of DNA has been proposed for α-synuclein, aggregation of which (in the form of Lewy bodies) is a pathological hallmark of PD and several other neurodegenerative disorders [65]. Recombinant α-synuclein has been found to cause DNA breaks *in vitro*, and its misfolding and oligomerization exacerbates damage even further [39]. Using a human neuroblastoma cell line, Vasquez *et al.* [39] went on to show that α-synuclein interacts with chromatin, and that the nuclear localization of α-synuclein results in an increase in DNA damage, as detected by an alkaline comet assay. The relevance for the disease is still unclear as increased DSB levels have, to our knowledge, not yet been reported in PD patients or in any of the other synucleinopathies.

Next to the presence of extracellular amyloid-β aggregates, AD is also associated with the formation of Tau aggregates [66]. Next to amyloid-β and α-synuclein, Tau has been suggested to influence genome integrity as well. Interestingly, the deletion of Tau in an AD mouse model characterized by elevated amyloid-β levels reduced *γ*-H2AX levels in neurons to wild-type control levels [31]. Such a response also occurred with Tau heterozygosity. This suggests that the detrimental effect of amyloid-β on DNA stability depends on the presence of Tau [31] and argues against direct DNA damage by fibrillar amyloid-β protein.

**Figure 2.**
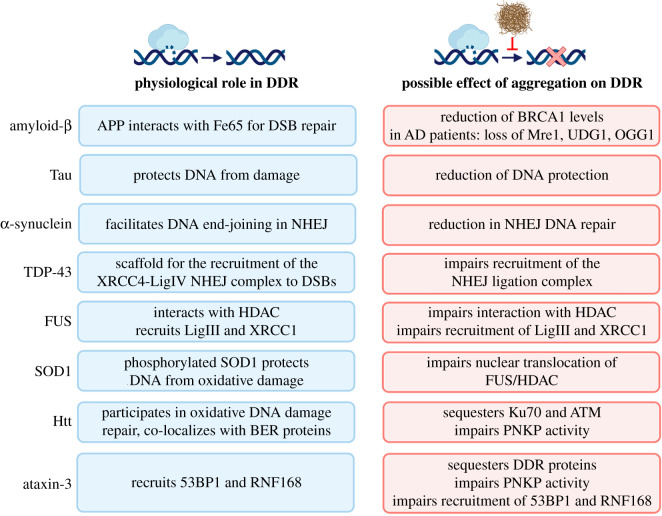
Neurodegenerative disease-associated proteins and their interactions with the DDR under physiological conditions (blue) and neurodegenerative pathological conditions (red). APP, amyloid precursor protein.

By contrast, other studies have reported that nuclear Tau protects the DNA from damage in cell culture under stress conditions [67] (figure 2). A recent *in vivo* study showed that Tau deficient mice without elevated amyloid-β have higher levels of DNA damage, detected with an alkaline comet assay in various parts of the brain including the cortex and hippocampus [34]. Additionally, AD-associated aggregation of Tau loses its ability to bind to DNA [35,36]; thus, it has been postulated that the loss of Tau from the nucleus could contribute to the accumulation of DNA damage as observed in AD patient brains. Alternatively, based on these results, it cannot be excluded that Tau has an active role in facilitating DNA repair.

### 2.2. Protein aggregation triggers oxidative stress and oxidative DNA damage

A major hallmark of neurodegeneration is oxidative stress [68]. Oxidative stress leads to mitochondrial dysfunction, which is present in various neurodegenerative diseases, and there are multiple mechanisms linking mitochondrial dysfunction and protein homeostasis [69]. Additionally, mitochondrial dysfunction itself results in increased levels of reactive oxygen species (ROS), creating a positive feedback loop of oxidative stress. The relationship between mitochondrial dysfunction, neurodegenerative diseases and ageing has been extensively reviewed [70–73].

As neurons already inherently generate high levels of ROS due to their active metabolic state [74], this has fuelled the hypothesis that ROS-mediated toxicity plays a central role in the pathophysiology of many neurodegenerative diseases. However, it is not entirely clear how oxidative stress relates to DNA damage and to a loss of protein homeostasis in this context. Here, we will focus on studies where changes in oxidative stress were observed in disease models in response to an accumulation of neurodegeneration-associated proteins.

The induction of protein aggregates triggers oxidative stress in several models, subsequently leading to oxidative DNA damage in neurons [24]. For example, mammalian neuronal AD cells treated with a toxic amyloid-β peptide fragment (amyloid-β_25−35_) showed increased ROS levels [32]. Additionally, in PD patients, oxidative DNA damage has been detected in the substantia nigra, as shown by elevated levels of 8-oxoguanine (8-oxoG) and the activation of DNA repair enzymes [42–44].

Other studies have found that α-synuclein also mediates an increase in DNA damage via oxidative stress. Injection of either cDNA encoding α-synuclein (using an AAV delivery system) or human α-synuclein pre-formed fibrils (PFF-syn) in the mouse substantia nigra and striatum increased DNA damage and activated the DDR [40]. Both AAV-syn- and PFF-syn-treated mice displayed α-synuclein aggregates, visible six and four months after injection, respectively, alongside increased levels of DNA damage, detected by staining for foci of DDR components γ-H2AX and 53BP1. In these *in vivo* models, protein aggregation often occurs relatively late, suggesting that it is not the protein aggregates themselves, but rather early, indirect mechanisms that underlie the increase in DNA damage. Indeed, PFF-syn-induced DNA damage and the activation of the DDR in the dopaminergic cell line SH-SY5Y were prevented by administration of the antioxidant N-acetylcysteine (NAC) [40], indicating that the elevated DNA damage observed in these PD models is occurring indirectly via oxidative stress induced by α-synuclein aggregates.

Oxidative stress is also thought to play a role in ALS [75], mostly via superoxide dismutase (SOD1), an enzyme that catalyses the breakdown of toxic superoxide radicals, which is associated with ALS [76]. A study of ALS patient blood cells expressing wild-type SOD1 but with high levels of cytoplasmic SOD1 aggregates revealed increased DNA damage, detected with an alkaline comet assay [46]. The opposite was true in ALS patient blood cells with nuclear SOD1 [46]. Furthermore, in human neuroblastoma cell lines, NLS-SOD1 rescued H_2_O_2_-induced DNA damage, whereas NES-SOD1 failed to do so [46]. This suggests that SOD1 activity is also required in the nucleus to protect DNA from oxidative damage and cytoplasmic aggregates compromise this activity. However, it is important to realize that most ALS-associated mutations, including those in SOD1, are dominant and always act in a background of a normal healthy allele [77]. A more recent study using an ALS mouse model expressing aggregation-prone mutant SOD1 reported an inverse correlation between the levels of aggregated SOD1 and disease progression, and found that a better marker for disease progression was the level of misfolded SOD1 [78]. Thus, it is postulated that misfolded SOD1 is responsible for the pathogenesis of ALS, whereas the formation of aggregates sequesters toxic misfolded proteins and may even be beneficial [78]. Oxidative stress may influence ALS indirectly as well. For example, the formation of stress granules is closely linked to ALS pathology and oxidative stress itself can trigger the formation of stress granules [79,80]. However, these links are rather indirect and have been discussed elsewhere [81].

Overall, it has been suggested that neurodegeneration-associated proteins (either soluble or as aggregate) can induce direct DNA damage. However, whether these proteins indeed cause DNA damage directly *in vivo* is less clear and most evidence points to an indirect role, for example, via the induction of oxidative damage. Alternatively, protein aggregation can also influence genome stability via inhibition of DDR pathways.

## 3. Protein aggregation impairs DNA repair pathways

Many studies have suggested that the aggregation of neurodegeneration-associated proteins can interfere with the DDR via several mechanisms (figure 1) [82]. Multiple distinct DNA repair pathways deal with different types of DNA damage (box 2), including non-homologous end joining (NHEJ), homologous recombination (HR) and base excision repair (BER) [13]. Here, we selected some examples that illustrate how defects in DNA repair pathways are associated with different aggregating protein species, and how they are affected in neurodegenerative disorders.

### 3.1. Alzheimer's disease: amyloid-β

Evidence of potential mechanisms linking protein aggregation and DSB repair capacity includes a decrease in NHEJ activity in post-mortem AD patient brain tissues, detected using a cell-free DNA end-joining assay [29]. Next to NHEJ, reduced neuronal HR has also been suggested in AD. The overexpression of amyloid-β in mice results in a reduction of BRCA1 in neurons, and the depletion of neuronal BRCA1 results in increased levels of DNA damage marker *γ*-H2AX in the hippocampus [83] (figure 2). The heterodimer BRCA1/BARD1 has E3 ligase activity and redirects DSB repair through HR, suggesting that a decreased level of BRCA1 reduces neuronal HR activity [84]. Furthermore, initial correlative studies of AD patient brain tissues showed a reduction in Mre11 compared with age-matched non-dementia controls [28]. Mre11, together with Rad50 and Nbs1, forms the MRN complex that recognizes DSBs, initiates DSB resection and triggers cell cycle checkpoint activation. Consequently, a reduction of Mre11 would cause both NHEJ and HR defects [85]. However, currently, it is unclear whether this decrease in BRCA1 or Mre11 protein levels are directly leading to reduced NHEJ or HR efficiency in the brain of AD patients. The reduced expression of these two core DSB repair proteins illustrates how protein aggregation by impeding protein expression may influence DNA repair capacity.

Similar to the reduced expression of DSB repair proteins, the reduced expression of BER components uracil-DNA glycosylase (UDG1) and 8-oxoguanine DNA glycosylase (OGG1) was also observed in AD patient brains, suggesting a decreased capacity to repair oxidative DNA damage [86] (figure 2). Furthermore, a correlation between reduced levels of BER components and incision activity, and an abundance of neurofibrillary tangles were found in patients with amnestic mild cognitive impairment (aMCI), a condition that is often observed in early AD progression [86]. However, no correlation was found between reduced BER and the number of amyloid-β plaques in this patient dataset [86]. Together these results suggest that deficiencies in BER activity occur early in AD progression, preventing the repair of oxidative damage and indirectly causing DNA damage. In line with this, reducing BER capacity in amyloid-β overexpressing mice exacerbates the AD-like phenotypes [87].

### 3.2. Parkinson's disease: α-synuclein

The physiological activity of α-synuclein has been proposed to play a more direct role in DNA repair. Knockout of α-synuclein in human cell culture inhibits DNA repair, and α-synuclein colocalizes with DSB repair components in response to bleomycin-induced DNA damage in human cell culture [41]. In the same study, an *in vitro* biochemical assay suggested that α-synuclein facilitates T4 ligase-mediated DNA end joining [41] (figure 2). A physiological role for α-synuclein in the nucleus has previously been inferred by a study in which neuronal cytoplasmic α-synuclein inclusions led to reduced levels of soluble α-synuclein in the nucleus [41,88]. Therefore, it is possible that these cytoplasmic inclusions result in pathologically low levels of α-synuclein in the nucleus, thereby reducing DSB repair capacity via its proposed role in NHEJ [41] (figure 2).

### 3.3. Amyotrophic lateral sclerosis: FUS, TDP-43 and SOD1

Changes to DSB repair mechanisms have also been observed in ALS models. For example, fused in sarcoma (FUS) is an RNA-binding protein that forms aggregates in ALS patients [89,90]. ALS patients with FUS mutations as well as FUS mutant mouse models display high levels of genome instability [49,50,91]. Whereas recombinant and endogenous FUS co-localizes to sites of radiation-induced DNA damage [92,93], the ALS-associated FUS mutant is unable to accumulate at sites of DNA damage [93]. Furthermore, ALS-associated FUS mutants have an impaired interaction with the chromatin remodeler histone deacetylase 1 (HDAC1), which plays a role in facilitating DNA repair [49,94,95]. This impaired interaction inhibits HDAC1 activity and therefore perturbs DSB repair [49]. The depletion of FUS resulted in reduced activity of both HR and NHEJ, suggesting that the involvement of FUS in DDR takes place upstream of these pathways [92]. However, it has also been postulated that FUS may also play a role in BER and SSB repair [93]. In the case of SSB repair, instead of an early function as in DSB repair, FUS facilitates the ligation of DNA nicks in motor neurons via recruitment of XRCC1 and LigIII to sites of DNA damage [48]. Additionally, aggregated ALS-associated mutant SOD1 has been shown to impair DNA repair in mouse motor neurons by inhibiting the translocation of the HDAC1/FUS complex to the nucleus [47].

TDP-43 is another RNA/DNA-binding protein that forms aggregates in ALS patients and models [96,97]. While TDP-43 is nuclear-localized under physiological conditions, it forms cytoplasmic aggregates in neurons of ALS patients [98]. The loss of TDP-43 from the nucleus in neuroblastoma SH-SY5Y cells results in increased levels of DSBs and DDR activity [45]. Furthermore, the loss of nuclear TDP-43 in ALS patients correlates strongly with TDP-43 aggregation and increased DNA damage [45]. This loss of genome stability is thought to be due to the reduction of NHEJ activity, as TDP-43 acts as a scaffold for recruitment of the XRCC4–LigIV NHEJ ligation complex in neural progenitor stem cells (NPCs) [45] (figure 2).

The aggregation of FUS, TDP-43 and SOD1 leads to reduced availability or impaired localization that limits their cellular function in genome maintenance, similarly to the proposed mechanism for the impact of α-synuclein aggregation on DSB repair [45,47,48]. In addition to the aggregation of these three infamous proteins, there are more links between ALS-associated genes and protein homeostasis. For example, senataxin (SETX), a helicase involved in the resolution of R-loops, is connected to autophagy [99]. SETX has also been linked to the ubiquitin-proteasome system (UPS), which plays a central role in PQC (box 1) [100,101]. Interestingly, SETX is also linked to the DDR [102]. In fact, several other ALS-associated genes such as NIMA-related kinase 1 (NEK1), p97 (also called VCP) and Ubiquilin 2 (UBQLN2) also have direct links to the DDR and proteostasis [103–110]. However, currently, it is unclear whether the mutant, ALS-associated, forms of these genes impede the DDR and if this influences the protein homeostasis.

### 3.4. Huntington's disease: Huntingtin

An alternative mechanism by which cytoplasmic aggregates can inhibit DDR is via the sequestration of DNA repair proteins. This has been observed in HD models; for example, HD-associated Huntingtin (Htt) mutant impairs DSB repair by sequestering the NHEJ-component Ku70 [51]. Mutant Htt was shown to colocalize and interact with Ku70, resulting in reduced Ku70 DNA-binding [51] (figure 2). Hence, the observed increased DNA damage in HD cellular and mouse models could be due to the impairment of DSB repair via NHEJ [51]. In a later study, the expression of polyQ inclusion bodies in human cells resulted in a failure to recruit 53BP1 to DSBs, where 53BP1 is known to redirect DSB repair through NHEJ, in line with a reduced NHEJ efficiency [63]. Moreover, mutant Htt sequesters the DDR kinase ATM in the cytoplasm, thereby contributing to reduced DSB repair in the nucleus [111]. Additionally, Htt co-localizes and interacts with BER proteins in response to oxidative stress [112].

HD-patient peripheral blood mononuclear cells also showed nuclear DNA damage due to a reduced activity of the BER pathway, demonstrated by reduced expression of BER components, including OGG1 [113]. Similarly, in transgenic HD cell and mouse models, mutant Htt impairs the function of the DNA repair enzyme polynucleotide kinase 3′-phosphatase (PNKP), disrupting BER-mediated DNA repair [114] (figure 2). These studies suggest that mutant Htt indirectly causes DNA damage by impairing BER activity. However, as mentioned above, the expression of aggregation-prone luciferase also impaired activation of the DDR [63]. Therefore, the loss of DDR in response to protein aggregation may not be specific to any particular protein, as it can also be caused by artificially created aggregates [63].

### 3.5. Spinocerebellar ataxia type 3: Ataxin-3

The sequestration of DNA repair proteins into Htt aggregates is similar to a mechanism observed in models of another polyQ protein, Ataxin-3, related to Machado-Joseph disease, also known as spinocerebellar ataxia type 3 (SCA3). Ataxin-3 is a deubiquitinating enzyme and expansions of the polyQ tract result in the formation of Ataxin-3 aggregates [115].

It has been claimed that the pathogenicity of mutant Ataxin-3 is due to the sequestration of DDR proteins in cytoplasmic inclusions [116,117]. For example, similar to mutant Htt, mutant aggregated Ataxin-3 inactivates the BER component PNKP in SCA3 cellular and mouse models, as well as in human brain samples [118]. Considering the persistent DNA damage also observed in these models, it is suggested that the formation of aggregates in SCA3 inhibits PNKP-mediated DNA repair mechanisms, causing genome instability [118]. Other studies pointed to the role of Ataxin-3 in DNA repair, independent of the expanded polyQ tract. The loss of Ataxin-3 in human cell cultures impaired the recruitment of DSB repair proteins 53BP1 and RNF168 to sites of DNA damage, and consequently lowered the levels of NHEJ and HR [119,120] (figure 2). These results show that the deubiquitinase activity of Ataxin-3 as part of the p97 hub is important for the DDR [119,120].

### 3.6. DNA repair proteins and aggregation

Strikingly, many DNA repair factors either directly aggregate or are sequestered into protein aggregates [118,121]. This indicates that the processes of protein aggregation and genome maintenance are somehow intrinsically connected. A possible explanation lies in the fact that, similar to most RNA-binding proteins, many DNA-binding proteins, including those involved in the DDR, rely heavily on regions of intrinsic disorder (IDRs) to perform their function [122,123]. These regions are thought to allow proteins to be structurally flexible and yet enable them to engage many different binding partners (i.e. proteins and nucleic acids) with high specificity [124].

Recently, a large body of evidence has shown that these IDRs also play an important role in catalysing liquid–liquid phase separation of proteins, thereby organizing them into membraneless biomolecular condensates [125]. By doing so, IDRs are fundamental to a wide range of cellular processes. In DNA repair, liquid–liquid phase separation is thought to enable the partitioning of relevant repair factors and simultaneously facilitate the required nucleic acid remodelling [126]. However, these same IDRs that are functionally so important also put proteins at the risk of aggregation. For liquid–liquid phase separation to occur, the local concentration of IDR proteins needs to be maintained at a very narrow bandwidth, just exceeding their solubility limit. As a result, biomolecular condensates are thought to be metastable, which is why they need to be regulated tightly to prevent aberrant phase transitions [127]. To this end, functional phase separation events are believed to rely heavily on the PQC network.

Situations of proteotoxic stress (i.e. where the regulatory capacity of the PQC network is insufficient) may lead to uncontrolled phase separation, causing condensates to irreversibly transition from a dense state to a solid aggregate. An example of this is TDP-43, which as described earlier is involved in RNA processing and DNA repair [128]. TDP-43 has a largely disordered C-terminal domain that drives liquid de-mixing under physiological conditions [129], a process thought to be regulated by molecular chaperones [130,131]. However, during stress, TDP-43 de-mixing can no longer be properly controlled, causing it to rapidly overshoot into an aggregated, proteotoxic state [129]. Similar molecular cascades may very well be responsible for the frequently observed sequestration of DNA repair factors discussed above into protein aggregates associated with neurodegeneration.

Additionally, there is evidence to suggest that amyloid precursor protein (APP), the precursor protein of amyloid-β aggregates, may also have a role in promoting DNA repair. In mammalian cell culture, APP interacts with the neuronal adaptor protein Fe65 to promote the chromatin remodelling required for the repair of DSBs (via histone acetyl transferase Tip60) [132] (figure 2). The C655F mutant of Fe65 (which is unable to bind to APP) cannot rescue the increased DNA damage observed in Fe65 deficient mouse embryonic fibroblasts [132]. This raises the possibility that the formation of cytoplasmic aggregates of amyloid-β might impair the physiological DDR repair the activity of APP.

## 4. DNA damage leading to loss of protein quality control

As discussed, protein aggregation-induced DNA damage is rapidly emerging as a contributing factor in the aetiology of many age-related neurodegenerative disorders. Importantly, the relationship between protein homeostasis and genome stability is a two-way street—aggregation of specific proteins like amyloid-β, Tau, mutant Htt and α-synuclein can impact genome stability, but conversely, genome instability can also dramatically affect the proteome [133]. This is clearly exemplified by known genetic alterations in more than 30 human genes that are strongly associated with protein aggregation and disease [134]. These alterations include (mostly missense substitution) mutations (e.g. in α-synuclein, Tau, transthyretin) and various nucleotide repeat expansions (ranging from CAG in HD and various SCAs, to C9ORF72 associated with ALS). In all these cases, protein homeostasis is threatened as a direct consequence of inherited or *de novo* genetic alterations in the germline, resulting in either the destabilization of native proteins, or in the formation of aberrant protein conformations that are prone to aggregate [134].

### 4.1. Gene copy number variation and protein stress

However, the connection between genome instability, loss of protein homeostasis and disease likely extends further. Larger chromosomal abnormalities can also result in potentially harmful transcript changes. For example, aneuploidy and gene copy number variations result in higher concentrations of protein that can drive proteotoxic stress [135–138]. This problem becomes especially pronounced when the genes involved encode components of stable multiprotein complexes relying on defined stoichiometries to fulfil their cellular function. Changes in the expression of any of these complex constituents can rapidly drive the excess of other components to aggregate, posing an added burden on the PQC network [139]. Although the proteome instability resulting from such dysregulated gene expression has been appreciated for years, it is gradually moving centre stage as a primary driver of dysfunction and disease [140].

### 4.2. DNA damage and protein stress

Recent single-cell sequencing studies have shown that a range of these ‘locked-in’ genetic alterations, including mutations and structural variants, can accumulate in somatic cells during ageing [141,142]. In addition, persistent global DNA damage can also challenge protein homeostasis by blocking transcription or, if bypassed, induce transcriptional mutagenesis, resulting in dysregulated gene expression or mutant protein production [143]. In line with a profound proteome-destabilizing impact of genome instability, the impairment of certain genome maintenance components has also been associated with proteome instability, although it is not always clear to what extent reduced DNA repair capacity is the causal factor. For example, the loss of the central DDR kinase ATM, involved in DSB repair, cell cycle regulation and cell death, leads to widespread protein aggregation, but this has been largely attributed to a signalling role in protein homeostasis, independent of genome maintenance itself [144]. More direct evidence supporting an impact of reduced genome maintenance comes from recent work showing that in mismatch repair-deficient tumours, the high mutation burden destabilizes the proteome, resulting in the accumulation of toxic protein aggregates that profoundly reduce cellular fitness [145]. How genome instability impacts global protein homeostasis over time, and to what extent this is relevant for disease and degeneration, is still poorly understood, but it is quickly surfacing as an extremely relevant field of research.

## 5. Future perspectives and conclusion

Ageing is characterized by both a loss of protein homeostasis and genome instability [146]. The brain is highly susceptible to both these events, as exemplified by the many neurodegenerative diseases hallmarked by the accumulation of protein aggregates, and by genetic DNA repair disorders characterized by pronounced neurodegeneration. Whether loss of protein homeostasis and genome instability are connected, and to what extent, remains unclear.

Recent evidence of a close relationship points towards increased DNA damage or reduced DNA repair capacity in the presence of neurodegeneration-associated proteins. However, there are still many key questions that remain unanswered. For example, it is not clear in which manner protein aggregates can directly cause DNA damage or indirectly impair the cellular DNA repair capacity. On the other hand, an increasing number of studies indicate that DNA damage might lead to a loss of protein homeostasis. It will therefore be important to determine if DNA damage has a causal role or is rather a collateral response of brain ageing; and/or, if a loss of protein homeostasis plays a role in the early stages of the disease or rather represents the end stage of a series of separate cellular responses, including DNA damage itself. To shed light on a possible causal relationship between the loss of protein homeostasis and DNA damage, a deeper understanding of the dynamic genome–proteome relationship is required. However, detailed proteogenomic characterization of somatic material is still in its infancy. The complexity of the brain and the low availability of patient post-mortem material in the early stages of pathology create an extra degree of difficulty. Nevertheless, promising recent advances in *in vitro* neuronal differentiation techniques and brain organoid cultures will aid in future progress. A combination of refined model systems and proteogenomic characterization will be needed to untangle the mechanisms that underlie the relationship between genotoxicity and proteotoxicity and how these responses contribute to neurodegeneration.

This review highlights that loss of protein homeostasis and genome instability contribute to neurodegeneration in a concerted manner. Understanding the underlying mechanisms connecting the PQC system and the DDR might lead to the identification of new therapeutic interventions that could benefit patients affected by neurodegenerative diseases and DNA repair disorders, as well as many cancer-surviving patients suffering from long-lasting adverse effects of DNA damage-inducing treatments.

## Abbreviations

53BP1: Tumour suppressor p53‐binding protein
AAV: Adeno-associated virus
AD: Alzheimer's disease
ALS: Amyotrophic lateral sclerosis
APP: Amyloid precursor protein
aMCI: Amnestic mild cognitive impairment
AOA1: Ataxia-oculomotor apraxia type 1
AT: Ataxia-telangiectasia
ATM: Ataxia-telangiectasia mutated
BARD1: BRCA1-associated RING domain protein 1
BER: Base excision repair
BRCA1: Breast cancer type 1 susceptibility protein
ChIP: Chromatin immunoprecipitation
CS: Cockayne syndrome
DDR: DNA damage response
DNA: Deoxyribonucleic acid
DSB: Double-strand break
EMSA: Electrophoretic mobility shift assay
Fe65: Protein encoded by APBB1 (amyloid beta precursor protein binding family B member 1)
FA: Fanconi anaemia
FUS: Fused in Sarcoma
HAP1: Human near haploid cells
HD: Huntington's disease
HDAC1: Histone deacetylase 1
HR: Homologous recombination
Htt: Huntingtin
IDR: Region of intrinsic disorder
iPSC: Induced pluripotent stem cell
KD: Knockdown
KO: Knockout
Ku70: Protein encoded by gene XRCC6 (X-ray repair cross complementing 6); Ku antigen, 70kDa
LigIII: DNA ligase 3
MMR: DNA mismatch repair
Mre11: Meiotic recombination 11
MRN: Mre11/Rad50/Nbs
mtDNA: Mitochondrial DNA
NAC: N-acetylcysteine
Nbs1: Nijmegen breakage syndrome 1
NEK1: NIMA-related kinase 1
NER: Nucleotide excision repair
NES: Nuclear export signal
NHEJ: Non-homologous end joining
NIMA: Never in mitosis, gene A
NLS: Nuclear localization signal
NPC: Neural progenitor stem cell
OE: Overexpression
OGG1: 8-oxoguanine DNA glycosylase
PBMC: Peripheral blood mononuclear cell
PD: Parkinson's disease
PFF: Pre-formed fibrils
PFF-syn: α-synuclein pre-formed fibrils
PNKP: Polynucleotide kinase 3′-phosphatase
polyQ: Polyglutamine
PQC: Protein quality control
RNA: Ribonucleic acid
RNF168: Ring finger protein 168
ROS: Reactive oxygen species
RQC: ribosome-associated protein quality control
SCA3: spinocerebellar ataxia type 3
scDNA: Supercoiled DNA
SETX: Senataxin
SOD1: Superoxide dismutase 1
SSB: Single-strand break
TDP-43: TAR DNA-binding protein 43
TUNEL: Terminal deoxynucleotidyl transferase dUTP nick end labelling
UBQLN2: Ubiquilin 2
UDG1: Uracil-DNA glycosylase
UPS: Ubiquitin-proteasome system
VCP: Valosin-containing protein
XP: xeroderma pigmentosum
XRCC1: X-ray repair cross-complementing protein 1
XRCC4: X-ray repair cross-complementing protein 4

## Data accessibility

This article does not contain any additional data.
